# *BRAF* Mutations in Advanced Cancers: Clinical Characteristics and Outcomes

**DOI:** 10.1371/journal.pone.0025806

**Published:** 2011-10-19

**Authors:** Hazem El-Osta, Gerald Falchook, Apostolia Tsimberidou, David Hong, Aung Naing, Kevin Kim, Sijin Wen, Filip Janku, Razelle Kurzrock

**Affiliations:** 1 Department of Investigational Cancer Therapeutics (Phase I Clinical Trials Program), The University of Texas MD Anderson Cancer Center, Houston, Texas, United States of America; 2 Department of Melanoma Medical Oncology, The University of Texas MD Anderson Cancer Center, Houston, Texas, United States of America; 3 Department of Biostatistics, The University of Texas MD Anderson Cancer Center, Houston, Texas, United States of America; Beth Israel Deaconess Medical Center, United States of America

## Abstract

**Background:**

Oncogenic *BRAF* mutations have been found in diverse malignancies and activate RAF/MEK/ERK signaling, a critical pathway of tumorigenesis. We examined the clinical characteristics and outcomes of patients with mutant (mut) *BRAF* advanced cancer referred to phase 1 clinic.

**Methods:**

We reviewed the records of 80 consecutive patients with *mutBRAF* advanced malignancies and 149 with wild-type (*wt*) *BRAF* (matched by tumor type) referred to the Clinical Center for Targeted Therapy and analyzed their outcome.

**Results:**

Of 80 patients with *mutBRAF* advanced cancer, 56 had melanoma, 10 colorectal, 11 papillary thyroid, 2 ovarian and 1 esophageal cancer. Mutations in codon 600 were found in 77 patients (62, *V600E*; 13, *V600K*; 1, *V600R*; 1, unreported). Multivariate analysis showed less soft tissue (Odds ratio (OR) = 0.39, 95%CI: 0.20–0.77, P = 0.007), lung (OR = 0.38, 95%CI: 0.19–0.73, p = 0.004) and retroperitoneal metastases (OR = 0.34, 95%CI: 0.13–0.86, p = 0.024) and more brain metastases (OR = 2.05, 95%CI: 1.02–4.11, P = 0.043) in patients with *mutBRAF* versus *wtBRAF*. Comparing to the corresponding *wtBRAF*, *mutBRAF* melanoma patients had insignificant trend to longer median survival from diagnosis (131 vs. 78 months, p = 0.14), while *mutBRAF* colorectal cancer patients had an insignificant trend to shorter median survival from diagnosis (48 vs. 53 months, p = 0.22). In melanoma, *V600K* mutations in comparison to other *BRAF* mutations were associated with more frequent brain (75% vs. 36.3%, p = 0.02) and lung metastases (91.6% vs. 47.7%, p = 0.007), and shorter time from diagnosis to metastasis and to death (19 vs. 53 months, p = 0.046 and 78 vs. 322 months, p = 0.024 respectively). Treatment with RAF/MEK targeting agents (Hazard ratio (HR) = 0.16, 95%CI: 0.03–0.89, p = 0.037) and any decrease in tumor size after referral (HR = 0.07, 95%CI: 0.015–0.35, p = 0.001) correlated with longer survival in *mutBRAF* patients.

**Conclusions:**

*BRAF* appears to be a druggable mutation that also defines subgroups of patients with phenotypic overlap, albeit with differences that correlate with histology or site of mutation.

## Introduction

The RAS proteins regulate cell proliferation, survival and differentiation by activating a number of downstream effectors, including RAF protein kinase. Once activated, RAF stimulates a signaling cascade involving the MEK/ERK pathway. BRAF, a serine-threonine kinase, is one of three RAF protein kinase family members (ARAF, BRAF and CRAF) [1]. The *BRAF* proto-oncogene has recently been the focus of intensive research, as its mutation constitutively activates RAF/MEK signaling, a major driver of carcinogenesis in various malignancies, most notably in melanoma, colon cancer, and papillary thyroid cancer^1^. The most common *BRAF* mutation is a substitution of glutamic acid for valine in codon 600 (V600E) [2]–[3].

In recent years, a plethora of promising compounds that target the RAS/RAF/MEK pathway have entered clinical trials, some of them demonstrating promising clinical activity, mainly in cancers with *BRAF* mutations [4]–[6]. Consequently, testing for activating mutations in *BRAF* is becoming more common, especially if patients are to be treated with BRAF inhibitors, or other pathway modulators such as MEK inhibitors.

Oncogenic mutations such as *BRAF* occur across diverse tumor types. Herein, we examined clinical features and outcome associated with the presence of *BRAF* mutations, with the main objectives being to outline clinical and prognostic characteristics associated with the presence of *BRAF* mutations, whether or not specific *BRAF* mutations have a distinct clinical course, as well predictive impact of targeted treatment.

## Methods

### Patients

Starting in January 2006, we investigated the *BRAF* mutation status of patients with advanced tumors and available tissue referred to the Clinical Center for Targeted Therapy in the Department of Investigational Cancer Therapeutics (Phase I Clinical Trials Program) at The University of Texas MD Anderson Cancer Center. The registration of patients in the database, pathology assessment, and mutation analysis were performed at MD Anderson. In total, 80 consecutive patients with *BRAF* mutations were selected.

To define distinguishing features of mutant (mut) *BRAF* advanced cancers, we selected a control group of consecutive patients with wild-type (wt) *BRAF* advanced cancers seen at our center during the same time period and matched in a 1∶2 ratio by tumor type with *mutBRAF* patients.

The MD Anderson Cancer Center Institutional Review Board has approved the study. Written consent was given by the patients for their information to be stored in the hospital database and used for research.

### Tissue samples and mutational analysis

Archival formalin-fixed, paraffin-embedded tissue blocks or material from fine-needle aspiration biopsy obtained from diagnostic and/or therapeutic procedures were used to test for *BRAF* mutations. All pathology was centrally confirmed at MD Anderson. *BRAF* mutation testing was performed in a Clinical Laboratory Improvement Amendment–certified Molecular Diagnostic Laboratory within the Division of Pathology and Laboratory Medicine at MD Anderson. DNA was extracted from micro-dissected paraffin-embedded tumor sections and analyzed using a polymerase chain reaction (PCR)-based DNA sequencing method for *BRAF* codons 595–600 mutations of exon 15 by pyrosequencing as previously described [7]. Substitution of glutamic acid for valine in codon 600 is denoted as V600E; V600K denotes substitution of lysine for valine; V600R, arginine for valine.

Whenever possible, we tested for other mutations such as *EGFR* (exons 18 and 21) [8], *KIT* (exons 11, 13 and 17) [9], *PIK3CA* (exons 9 and 20) [10], *NRAS* and *KRAS* (exon 2) [7], [11]. PTEN loss was assessed using immunohistochemistry (monoclonal mouse anti-human PTEN, clone 6H2.1, Dako®, Denmark) [12].

### Clinical characteristics and treatment evaluation

All clinical variables were assessed by review of the electronic medical record. Treatment efficacy was evaluated from computed tomography (CT) scans and/or magnetic resonance imaging (MRI) at baseline before treatment initiation and then about every 6 to 8 weeks. All radiographs were read in the Department of Radiology at MD Anderson and reviewed in the Department of Investigational Cancer Therapeutics tumor measurement clinic.

Prognostic assessment was done using the Royal Marsden Hospital (RMH) [13] prognostic score as follows: 0 points, normal lactate dehydrogenase (LDH), albumin ≥3.5 g/dL, a ≤2 metastatic sites; 1 point- LDH>upper limit of normal, albumin <3.5 g/dL, >2 metastatic sites. Patients with 0–1 points had a good RMH score, and patients with 2–3 points had a poor RMH score.

### Statistical Analysis

Statistical analysis was verified by our statistician (SW). The following covariates pertaining to patient characteristics were analyzed: type of cancer, age, gender, race, personal history of cancer, history of smoking or alcoholism, family history of cancer, site and number of metastases, presence of ascites, pleural effusion or deep venous thrombosis, tumor markers (CEA, CA 19-9, CA125, CA27.29), lactate dehydrogenase, albumin, hemoglobin, white blood cell count, platelet count, calcium level, site of mutation, presence of other aberrations (*PIK3CA*, *NRAS or KRAS*, *KIT* mutation and *PTEN* loss), date of diagnosis, locally advanced disease, distant metastases, referral, death or date of last follow-up, information about best standard systemic treatment for metastatic disease and treatment with phase 1 trial.

Descriptive statistics were used to summarize patient characteristics. The Fisher exact test was used to test for any association between two categorical variables. Mann-Whitney U test was used to test for association between age and *BRAF* mutation status.

Overall survival (OS) was measured (method of Kaplan-Meier) from the time of diagnosis, date of metastases, or date of referral to the date of death or last follow-up, whichever occurred first. Patients alive were censored at the last follow-up date. Progression-free survival (PFS) was defined as the time interval between the start of therapy to the first observation of disease progression (as determined by Response Evaluation Criteria in Solid Tumors (RECIST) [36] or death, whichever came first. Patients alive and without disease progression were censored at the last follow-up date. Disease-free survival was measured from time of diagnosis to first distant metastases. Log-rank test was used to compare OS or PFS among subgroups. Multivariate analysis with the Cox proportional hazards regression model was used to assess an independent association between a characteristics and PFS or OS. The “enter” method was used where all the variables are entered in the model without checking. Binary logistic regression method was used to test for any independent correlation between a categorical variable and *BRAF* mutational status. The “enter” method was used where all the variables are entered in the model without checking. All tests were two-sided. A p value less than 0.05 was considered statistically significant. Statistical analysis was performed using SPSS (version 17.0; SPSS, Chicago, IL, USA).

## Results

### Patient characteristics

A total of 80 patients with advanced tumors and *mutBRAF* were identified. The median age was 52 years (range, 18–78 years), and 43 were men (54%). The majority of patients had melanoma (n = 56, 70%) followed by papillary thyroid carcinoma (n = 11, 14%), colorectal cancer (n = 10, 13%), and other tumor types (ovarian cancer, n = 2, 2%; esophageal cancer, n = 1, 1%) (reflecting referral patterns to our clinic), (Table 1).

**Table 1:** Clinical characteristic of 80 patients with *BRAF*-mutant disease and 149 matched controls with *BRAF*-wild-type (Univariate Analysis).

	*mutBRAF* (N = 80)	*wtBRAF* (N = 149)	P value
**Age at diagnosis (median, range)**	52 (18–78)	58 (24–87)	**0.002**
**Age at diagnosis ≥60 years**	27 (34%)	69 (46%)	**0.07**
**Gender**			
**Men**	43 (54%)	100 (67%)	**0.06**
**Women**	37 (46%)	49 (33%)	**0.06**
**Race**			
**Caucasian**	67 (87%)	130 (87%)	Not significant
**Hispanic**	8 (10%)	9 (6%)	Not significant
**Asian**	2 (3%)	1 (1%)	Not significant
**African-American**	0 (0%)	9 (6%)	Not significant
**Type of Cancer**			
**Melanoma**	56 (70%)	112 (75%)	Not significant
**Colorectal cancer**	10 (13%)	20 (14%)	Not significant
**Papillary thyroid cancer**	11 (14%)	11 (7%)	Not significant
**Ovarian cancer**	2 (2%)	4 (3%)	Not significant
**Esophageal cancer**	1 (1%)	2 (1%)	Not significant
**Personal history of cancer**	15 (19%)	27 (18%)	0.99
**Family history of cancer**	62 (78%)	120 (81%)	0.61
**First degree**	48 (60%)	96 (64%)	0.56
**Age<60 years**	25 (31%)	43 (29%)	0.76
**First degree & age<60**	16 (20%)	30 (20%)	0.99
**Social history**			
**Tobacco**	26 (33%)	61 (41%)	0.25
**Alcohol**	11 (14%)	28 (19%)	0.36
**Site of metastasis**			
**Brain**	27 (34%)	45 (30%)	0.65
**Liver**	31 (39%)	67 (45%)	0.40
**Lung**	48 (60%)	118 (79%)	**0.003**
**Retroperitoneum**	9 (11%)	37 (25%)	**0.004**
**Bone**	20 (25%)	41 (28%)	0.75
**Superficial Lymph Node**	39 (49%)	76 (51%)	0.78
**Soft tissue**	26 (33%)	75 (50%)	**0.01**
**Peritoneum**	33 (41%)	61 (41%)	0.99
**Mediastinum**	19 (24%)	38 (26%)	0.87
**Stage at diagnosis**			
**Early stage**	57 (71%)	91 (61%)	0.14
**Locally advanced stage**	13 (16%)	36 (24%)	0.18
**Metastatic stage**	10 (13%)	22 (15%)	0.69
**Pleural effusion**	11 (14%)	17 (11%)	0.60
**Ascites**	8 (10%)	13 (9%)	0.74
**Thrombosis**	15 (19%)	30 (20%)	0.80
**Site of mutation**			
**C600/599**	1 (*V600E/T599S*)	N/A	N/A
**C600**	77 ( 62 *V600E*, 13 *V600K*, 1 *V600R*, 1 unknown)	N/A	N/A
**C601**	2 (2 *K601E*)	N/A	N/A
**PTEN loss**	2/7^1^ (29%)	2/20 (10%)	0.27
***KRAS*** ** mutation**	0/24 (0%)	13/45 (29%)	**0.002**
***PIK3CA*** ** mutation**	1/26 (4%)	4/46 (9%)	0.64
***NRAS*** ** mutation**	1/17 (6%)	42/108 (39%)	**0.006**
***KIT*** ** mutation**	0/30 (0%)	3/93 (3%)	0.99
***EGFR*** ** mutation**	0/18 (0%)	0/43 (0%)	0.99
**Median time from diagnosis to metastases (months) (95%CI)**			
**Melanoma**	44 (17–71)	20 (16–24)	**0.058**
**Colorectal cancer**	0	8 (0–28.4)	0.96
**Papillary thyroid cancer**	37 (0–74.7)	73 (29.8–116.1)	0.45
**Combined**	28 (12.8–43.1)	19 (14.5–23.5)	0.13
**Time from diagnosis to metastasis ≥2 years**	45 (56%)	63 (42%)	**0.052**

1 Denominator refers to the number of patients tested.

The most common metastatic sites were lungs (n = 48, 60%), superficial lymph nodes (n = 39, 49%), peritoneum (n = 33, 41%), liver (n = 31, 39%), brain (n = 27, 34%), soft tissue (n = 26, 33%), bones (n = 20, 25%) and retroperitoneal lymph nodes (n = 9, 11%).

We identified 149 control patients with advanced cancers who tested negative for *BRAF* mutations in the same time period and who were matched on a 1∶2 basis by tumor type with *mutBRAF* patients. For papillary thyroid cancer, matching was done with a 1∶1 ratio due to an inadequate number of patients referred who had tests done and tested negative for *BRAF* mutation. The detailed patient characteristics are shown in Table 1.

Groups with *mutBRAF* and *wtBRAF* were similar in terms of median time from diagnosis to referral to the phase 1 clinic as calculated by log-rank method (12 vs. 12.7 months, p = 0.95). Initial cancer staging at diagnosis was also equally distributed among the two groups. Patients were treated on a clinical trial if they had failed to respond to conventional treatment. Whenever possible, patients with *mutBRAF* were offered treatment targeting the RAF/MEK pathway. Patients had a median of two prior treatments, regardless of BRAF status.

### Types of *BRAF* mutations

Of the 80 patients with *mutBRAF*, 77 (96%) had mutations in codon 600 and two (3%) in codon 601. One (1%) patient had simultaneous mutations in codons 599 and 600. Of the 77 patients with codon 600 *mutBRAF*, 62 (81%) had *V600E* mutations (melanoma, n = 40; colorectal, n = 8; papillary thyroid cancer, n = 11; esophageal, n = 1 and ovarian, n = 2), 13 (17%) *V600K* mutations (melanoma, n = 12; colorectal cancer, n = 1), 1 (1%) *V600R* mutation (melanoma, n = 1) and one of unreported type (colorectal, n = 1) (Table 1).

### *BRAF* mutations and clinical features

#### Univariate Analysis

Patient age at diagnosis was significantly younger for patients with *mutBRAF* (median age = 52 years) versus *wtBRAF* disease (median age = 58 years) (p = 0.002). Men were more commonly represented in both *mutBRAF* and *wtBRAF* groups, but the proportion of women trended towards being greater in the *mutBRAF* group (46% vs. 33%, p = 0.06). There were no significant differences between the *mutBRAF* and *wtBRAF* group for other characteristics, including ethnicity, personal, social and family history, complications including thrombosis, ascites and pleural effusion (Table 1).

Patients who had *mutBRAF* tumors had less frequent involvement of the lungs (60% vs. 79%; p = 0.003), retroperitoneal nodes (11% vs. 25%; p = 0.004), and soft tissue (33% vs. 50%; p = 0.01). In subgroup analysis, this pattern was also observed in each of the three major tumor types; however due to the small number of patients in the non-melanoma cohort, significance was only achieved for patients with melanoma (unshown data). There was no difference in involvement of other sites by metastases.

#### Multivariate Analysis

In multivariate analysis using a logistic regression model, patients with *mutBRAF* had less frequent metastases to (i) soft tissue (OR = 0.39, 95% CI: 0.20–0.77, p = 0.007); (ii) lung (OR = 0.38, 95% CI: 0.19–0.73, p = 0.004); and (ii) the retroperitoneum (OR = 0.34, 95% CI: 0.13–0.86, p = 0.024) (Table 2). Women were more likely to have *mutBRAF than wtBRAF* (OR = 1.92, 95% CI: 1.02–3.57, p = 0.045). Patients with *mutBRAF* compared with *wtBRAF* were more likely to have brain metastases (OR = 2.05, 95% CI: 1.02–4.11, p = 0.043). Patients younger than 60 years showed a trend towards higher likelihood of *BRAF* mutations (OR = 1.88, 95% CI: 0.99–3.70, p = 0.053). In subgroup analysis of melanoma, this trend was statistically significant (multivariate p value = 0.023) (Table 3). The smaller numbers of patients with other cancers precluded a separate analysis for this factor. An interval from diagnosis to distant metastases of ≥2 years was more likely to be associated with *mutBRAF* (Odds ratio (OR) = 2.84, 95% Confidence interval (CI): 1.18–4.14, p = 0.013) (Table 2). However, in disease specific analyses, in colorectal and papillary thyroid cancer, the proportion of patients with a disease-free interval from diagnosis to metastases of over two years was less for patients with *mutBRAF* disease, but this did not reach statistical significance because of the small number of patients in each subgroup (data not shown).

**Table 2:** Multivariate analysis by logistic regression model showing the clinico-pathological features correlated with the *BRAF* mutation.

Clinical feature	Odds Ratio	Lower 95%	Upper 95%	P value
**Age<60 years**	1.88	0.99	3.70	0.053
**Women**	1.92	1.02	3.57	**0.045**
**Metastatic site**				
**Soft tissue**	0.39	0.20	0.77	**0.007**
**Brain**	2.05	1.02	4.11	**0.043**
**Lung**	0.38	0.19	0.73	**0.004**
**Liver**	0.86	0.46	1.63	0.665
**Retroperitoneum**	0.34	0.13	0.86	**0.024**
**Bone**	1.10	0.53	2.26	0.78
**Peritoneum**	0.97	0.51	1.83	0.92
**Superficial lymph node**	0.91	0.47	1.75	0.79
**Time from diagnosis to metastasis ≥2 years**	2.21	1.18	4.15	**0.013**

**Table 3:** Multivariate analysis by logistic regression model showing the clinico-pathological features correlated with the *BRAF* mutation in melanoma patients.

Clinical feature	Odds Ratio	Lower 95%	Upper 95%	P value
**Age<60 years**	2.57	1.13	5.81	**0.023**
**Women**	2.27	1.01	5.12	**0.047**
**Metastatic site**				
**Soft tissue**	0.36	0.16	0.83	**0.017**
**Brain**	2.37	1.05	5.37	**0.038**
**Lung**	0.28	0.12	0.63	**0.002**
**Liver**	0.93	0.41	2.08	0.85
**Retroperitoneum**	0.32	0.11	0.95	**0.04**
**Bone**	1.39	0.57	3.42	0.46
**Peritoneum**	0.78	0.34	1.78	0.56
**Superficial lymph node**	0.74	0.34	1.62	0.45
**Time from diagnosis to metastasis ≥2 years**	2.96	1.36	6.45	**0.006**

### Co-Existing Mutations/Molecular Aberrations

A subset of *mutBRAF* patients with available data also had *PTEN* loss (2/7; 29%) or *PIK3CA* mutations (1/26; 4%) (Table 1). In patients with *wtBRAF*, 2/20 (10%) had *PTEN* loss and 4/46 (9%) had PIK3CA mutation. There was no difference in the rates of *PTEN* loss or *PIK3CA* mutations between *mutBRAF* vs. *wtBRAF* groups, but the small numbers of patients may preclude firm conclusions, especially in the PTEN group.

As expected *KRAS* and *NRAS* mutations were significantly less common in the *mutBRAF* group compared to the *wtBRAF* group (*KRAS*: 0/24 (0%) vs. 13/45 (29%), p = 0.002; *NRAS*: 1/17 (6%) vs. 42/108 (39%), p = 0.006). Of interest, it should be noted that one patient had a concomitant *BRAF* and *NRAS* mutation.

### *BRAF* status and Progression-free survival (PFS) on conventional standard treatment

We analyzed PFS on conventional treatment (before referral to phase 1 clinic) for metastatic disease according to *BRAF* status. We chose the longest PFS each patient had ever achieved on a conventional treatment.

**Figure 1:**
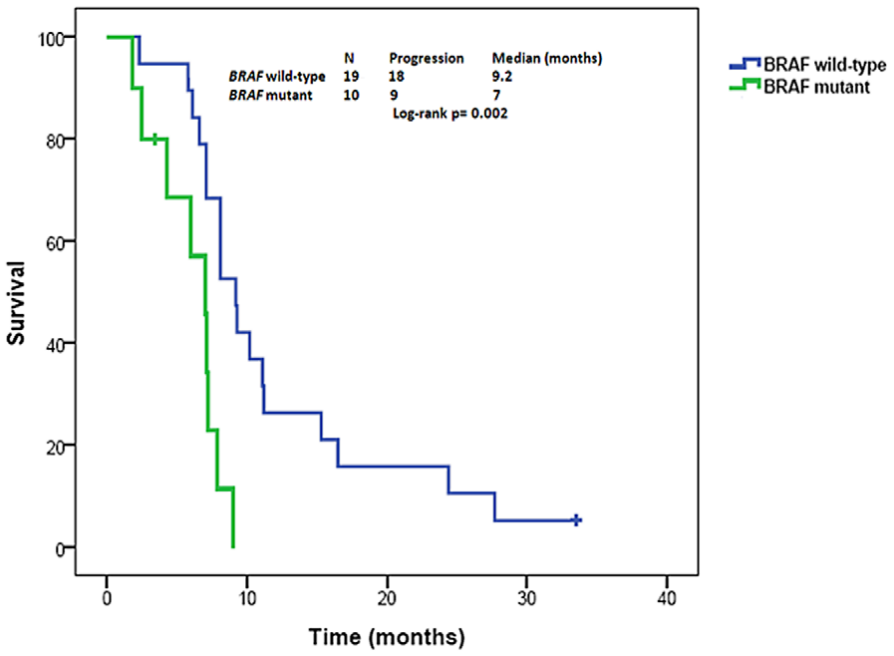
Kaplan Meier curve showing progression-free survival on best standard systemic treatment comparing patients with *mutBRAF* vs. *wtBRAF* metastatic colorectal cancer. (One patient with inadequate records on prior treatment was excluded).

When analyzed with all patients included, there was no overall difference in median PFS between *mutBRAF* vs. *wtBRAF* disease (7.0 months, 95%CI 5.6–8.3 vs. 7.1 months, 95%CI 5.7–8.5; p = 0.49). However, patients with colorectal cancer and *mutBRAF* had a median PFS of 7 months (95%CI 5.3–8.6) compared to 9.2 months (95%CI 7.4–10.9) in *wtBRAF* (p = 0.002) (Figure 1). In multivariate analysis, *mutBRAF* was an independent prognostic factor for shorter PFS (HR: 3.76, 95% CI 1.22–11.49, p = 0.02) on the best standard systemic therapy in metastatic colorectal cancer.

In melanoma and papillary thyroid cancer, there was no difference in median PFS in patients with *mutBRAF* compared to *wtBRAF* (4.3 months, 95%CI 1.9–6.8 vs. 5.5 months, 95%CI 3.5–6.7; p = 0.29; 24 months, 95%CI 14.4–33.5 vs. 25 months, 95%CI 0–55.4; p = 0.65 respectively).

### *BRAF* Status and Survival

#### Univariate Analysis

We analyzed OS from time of diagnosis and from time of metastasis. The median OS from time of diagnosis of *mutBRAF* patients was 322 months vs. 112 months (95%CI 58.2–165.7) for *wtBRAF* patients (p = 0.24). The median OS from time of metastasis of *mutBRAF* patients compared to *wtBRAF* was 99 months (95%CI 17.1–180.8) vs. 51 months (95%CI 38.7–63.2) (p = 0.58).

In disease specific subgroup analysis, the median OS from diagnosis and from metastasis was numerically longer in melanoma patients with *mutBRAF* compared to *wtBRAF* (131 months 95%CI 52.7–209.2 vs. 78 months, 95%CI 41.8–114.1; p = 0.14 and 35 months 95%CI 8.7–61.2 vs. 30 months, 95%CI 8.3–53.6; p = 0.63 respectively). In contrast, in colorectal cancer, the median OS from diagnosis and from metastasis was numerically shorter in *mutBRAF* patients compared to *wtBRAF* (48 months 95%CI 23.4–72.5 vs. 53 months, 95%CI 0–125.2; p = 0.22 and 30 months, 95%CI 14.5–45.4 vs. 53 months, 95%CI 38.8–67.1; p = 0.26 respectively). Small number of patients in disease specific subgroups precluded more definite conclusions and might explain the lack of statistical significance. The OS from time of diagnosis and metastasis did not differ between *mutBRAF* and *wtBRAF* patients with papillary thyroid cancer. The median OS from time of diagnosis was not reached after a follow-up of 133 and 138 months for *mutBRAF* and *wtBRAF* respectively. Also, the median OS from metastases was not reached with a median follow-up of 67 and 46 months respectively.

Further, we analyze the prognostic significance of *NRAS* in melanoma by stratifying our melanoma patients as follows: *mutBRAF*/*wtNRAS*, *wtBRAF/mutNRAS*, and *wtBRAF/wtNRAS*. A median OS from diagnosis in each of the 3 groups was 131 months (95%CI 81.6–180.3) (*mutBRAF*/*wtNRAS*), 67 months (95%CI 29–105) (*wtBRAF/mutNRAS*), and 109 months (95%CI 51.6–166.3) (*wtBRAF/wtNRAS*).The OS difference between *mutBRAF*/wt*NRAS* and *wtBRAF/mutNRAS* was of borderline statistical significance (p = 0.05). A median OS from time of metastasis was 35 months (95%CI 8.5–61.5), 20 months (95%CI 10.3–29.6), and 51 months (95%CI 4.8–97.1), respectively (p = 0.45). These data suggest that patients with *mutBRAF* melanoma survive longer than those with *NRAS-*mutant disease, but that the survival of *mutBRAF* melanoma is not different from that of melanoma patients with *wtBRAF* and *wtNRAS*.

#### Multivariate analysis

A multivariate analysis on all 229 patients based on age, gender, *RAS* (*KRAS*, *NRAS*) mutations, *BRAF* mutations, and disease type was conducted to determine whether any of these factors affects survival. *NRAS* mutation and male gender were the only independent factors associated with shorter OS from time of diagnosis (Hazard ratio (HR): 2.52, 95%CI 1.32–4.80, p = 0.005 and HR: 2.84, 95%CI 1.46–5.53, p = 0.002, respectively) whereas diagnosis of melanoma predicted a better OS from time of diagnosis (HR: 0.15, 95%CI 0.04–0.58, p = 0.005). Male gender was the only factor predicting poor OS from time of metastasis (HR: 2.79, 95%CI 1.42–5.45, p = 0.003).

A disease-specific multivariate analysis including age, gender, *RAS* (*KRAS*, *NRAS*) mutations and *BRAF* mutations was performed. In melanoma, only *NRAS* mutation and male gender were associated with shorter OS from time of diagnosis (HR: 2.16, 95% CI 1.11–4.18, p = 0.02 and HR: 2.64, 95% CI 1.28–5.41, p = 0.008, respectively). Male gender was the only prognostic factor for shorter OS from time of metastasis (HR: 2.84, 95% CI 1.35–5.97, p = 0.006). In colorectal cancer, only *KRAS* mutation was identified as an independent indicator for poor OS from time of diagnosis and metastasis (HR: 13.56, 95% CI 1.61–113.88, p = 0.016 and HR: 5.46, 95% CI 1.07–27.89, p = 0.04 respectively). We also detected a trend for *mutBRAF* to predict poor OS from diagnosis or first time of metastasis (HR: 8.31, 95% CI 0.95–72.56, p = 0.055 and HR: 4.05, 95% CI 0.75–21.76, p = 0.10, respectively).

In multivariate analysis, no prognostic factor was detected for papillary thyroid carcinoma, perhaps due to the low number of cases.

### Survival in the Clinical Center for Targeted Therapy (phase I clinic) according to the *BRAF* status

We performed a univariate and multivariate analysis to examine the factors that might predict OS from time of referral to the Clinical Center for Targeted Therapy (Phase I Program) until death in *mutBRAF* patients. Factors included were: age (≥60 vs. <60 years, gender (male vs. female), tumor type, RMH prognostic score, age, Eastern Cooperative Oncology Group (ECOG) performance status (0–1 vs. ≥2), treatment with RAF/MEK targeting agents vs. never treated with RAF/MEK targeting agent, any decrease in target lesion size vs. no decrease after referral to the phase 1 trial.

#### Univariate analysis in *mutBRAF* patients

**Figure 2:**
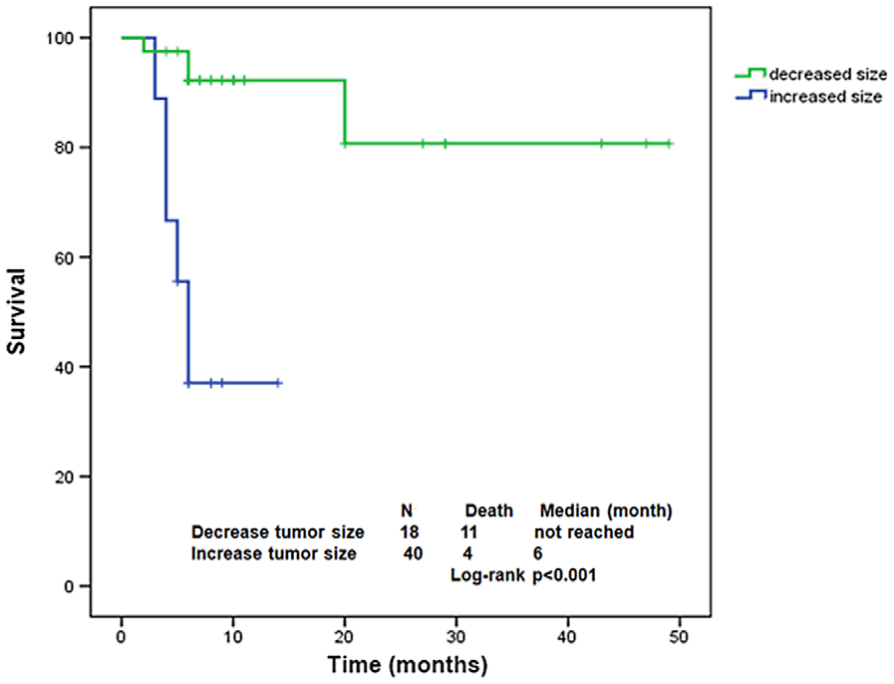
Kaplan-Meier estimate of overall survival from time of referral to phase 1 clinic in patients with *BRAF* mutation who showed any decrease vs. no decrease in size of target lesions on phase 1 trial. (Patients who did not have tumor measurements at the time of last follow-up (N = 9) or patients who were not enrolled in a phase 1 trial after referral (N = 13) were excluded).

In univariate analysis, we observed a longer OS from referral in women vs. men (not reached in both groups, p = 0.015 and HR 2.62, 95%CI 1.14–6.01; p = 0.02), RMH score of 0–1 vs. 2–3 (not reached vs. 5 months, 95%CI 3–7; p<0.001 and HR 3.69, 95%CI 1.74–7.82; p = 0.001), performance status ≤1 vs. 2–4 (not reached vs. 6 months, 95%CI 2.1–9.9; p = 0.035 and HR 2.51, 95%CI 1.01–6.28; p = 0.048), treatment with RAF/MEK targeting agents (56 of the 80 patients received RAF/MEK targeting agents including 37 with melanoma, 10 with papillary thyroid, 8 with colon cancer and 1 with ovarian cancer) vs. treatment with any other agents or no treatment (not reached vs. 5 months, 95%CI 3.4–6.6; p<0.001 and HR 0.20, 95%CI 0.095–0.43; p<0.001), papillary thyroid cancer vs. other cancers (not reached in both groups, p = 0.018 and HR 0.09, 95%CI 0.10–0.89; p = 0.04), and any decrease in tumor size on any phase I clinical trial vs. no decrease (not reached vs. 6 months, 95%CI 4.7–7.2; p<0.001 and HR 0.09, 95%CI 0.025–0.32; p<0.001) (Figure 2 and Table 4).

**Table 4:** Univariate analysis of survival predictors after referral to phase 1 clinic in patients with *mutBRAF* advanced cancer.

Predictor	Median OS (95% CI)	N	P value	HR	95%CI	P value
**Age≥60**	Unreached	36	0.57	1.23	0.58–2.58	0.58
**Age<60**	Unreached	44				
**Male**	Unreached	43	**0.015**	2.62	1.14–6.01	**0.02**
**Female**	Unreached	37				
**RMH score** 1 ** 2–3**	5 (3–7)	57	**<0.001**	3.69	1.74–7.82	**0.001**
**RMH score 0–1**	Unreached	23				
**Performance status >1**	6 (2.1–9.9)	11	**0.035**	2.51	1.01–6.28	**0.048**
**Performance status 0–1**	Unreached	69				
**RAF/MEK targeting agents**	Unreached	56	**<0.001**	**0.20**	**0.095–0.43**	**<0.001**
**Other** 2	5 (3.4–6.6)	24				
**Brain metastasis**	Unreached	27	0.08	1.90	0.89–4.05	0.09
**No Brain metastasis**	Unreached	53				
**Time from diagnosis to metastases ≥2 years** 3	Unreached	45	0.36	0.71	0.33–1.51	0.38
**Time from diagnosis to metastases <2 years**	Unreached	34				
**Melanoma**	Unreached	56	0.38	1.46	0.60–3.57	0.39
**Non melanoma**	Unreached	24				
**Colorectal cancer**	5 (2.1–7.9)	10	0.11	2.13	0.80–5.69	0.12
**Non Colorectal cancer**	Unreached	70				
**Papillary thyroid cancer**	Unreached	11	**0.018**	0.09	0.10–0.89	**0.04**
**Non papillary thyroid cancer**	Unreached	69				
**Any decrease tumor size** 4	Unreached	40	**<0.001**	0.09	0.025–0.32	**<0.001**
**Any increase tumor size**	6 (4.7–7.2)	18				

1 Royal Marsden Hospital (RMH) ^13^ prognostic score is determined as follows: 0 points, normal lactate dehydrogenase (LDH), albumin ≥3.5 g/dL, a ≤2 metastatic sites; 1 point- LDH>upper limit of normal, albumin <3.5 g/dL, >2 metastatic sites. Patients with 0–1 points had a good RMH score, and patients with 2–3 points had a poor RMH score.

2 Includes patients treated with other agents (N = 11) as well as patients who never started on phase 1 trial (N = 13).

3 One patient of whom the exact date of diagnosis was not documented was excluded only from the univariate analysis comparing the OSref between patients who had a time from diagnosis to metastasis less or more than 2 years.

4 Patients who never had a restaging at the last follow-up or who never started on a phase 1 trial were excluded in the univariate analysis (N = 22).

**Figure 3:**
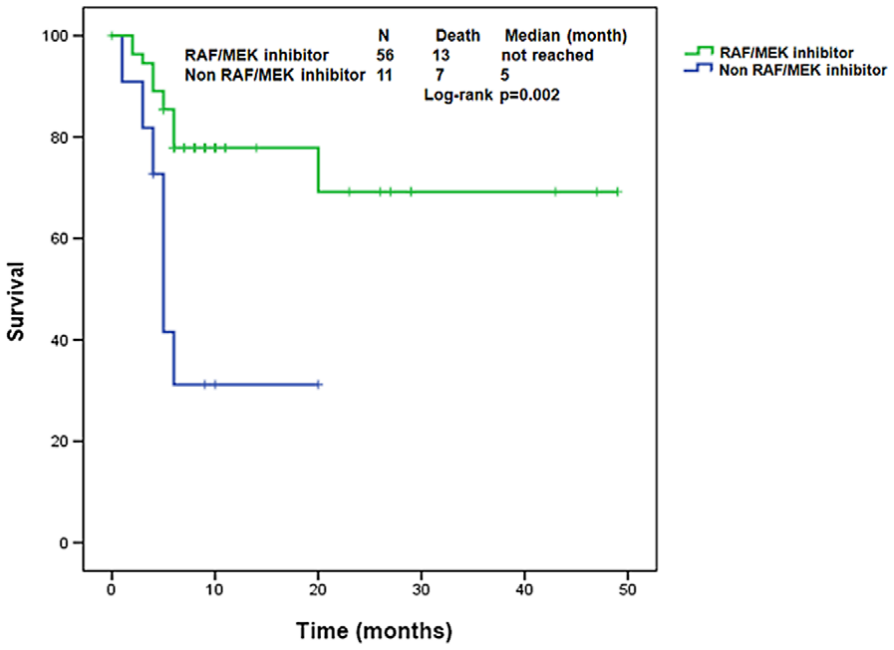
Kaplan-Meier estimate of overall survival from time of referral to phase 1 clinic in patients with *mutBRAF* treated with RAF/MEK targeting agents or other phase 1 trials. Tic marks represent patients still alive at the last follow-up. (Of 80 patients with *BRAF* mutations, 56 received a RAF/MEK targeting agents, 11 received a non RAF/MEK targeting agents and 13 were not enrolled on a phase 1 trial).

By excluding patients who did not get enrolled into a phase 1 trial after referral (13 patients total), we found that *mutBRAF* patients treated with RAF/MEK targeting agents has improved survival after referral compared to *mutBRAF* patients treated with any other agents (not reached vs. 5 months, 95%CI 4–6; p = 0.002 and HR 0.26, 95%CI 0.10–0.66; p = 0.005) (Figure 3).

#### Multivariate analysis in *mutBRAF* patients

**Figure 4:**
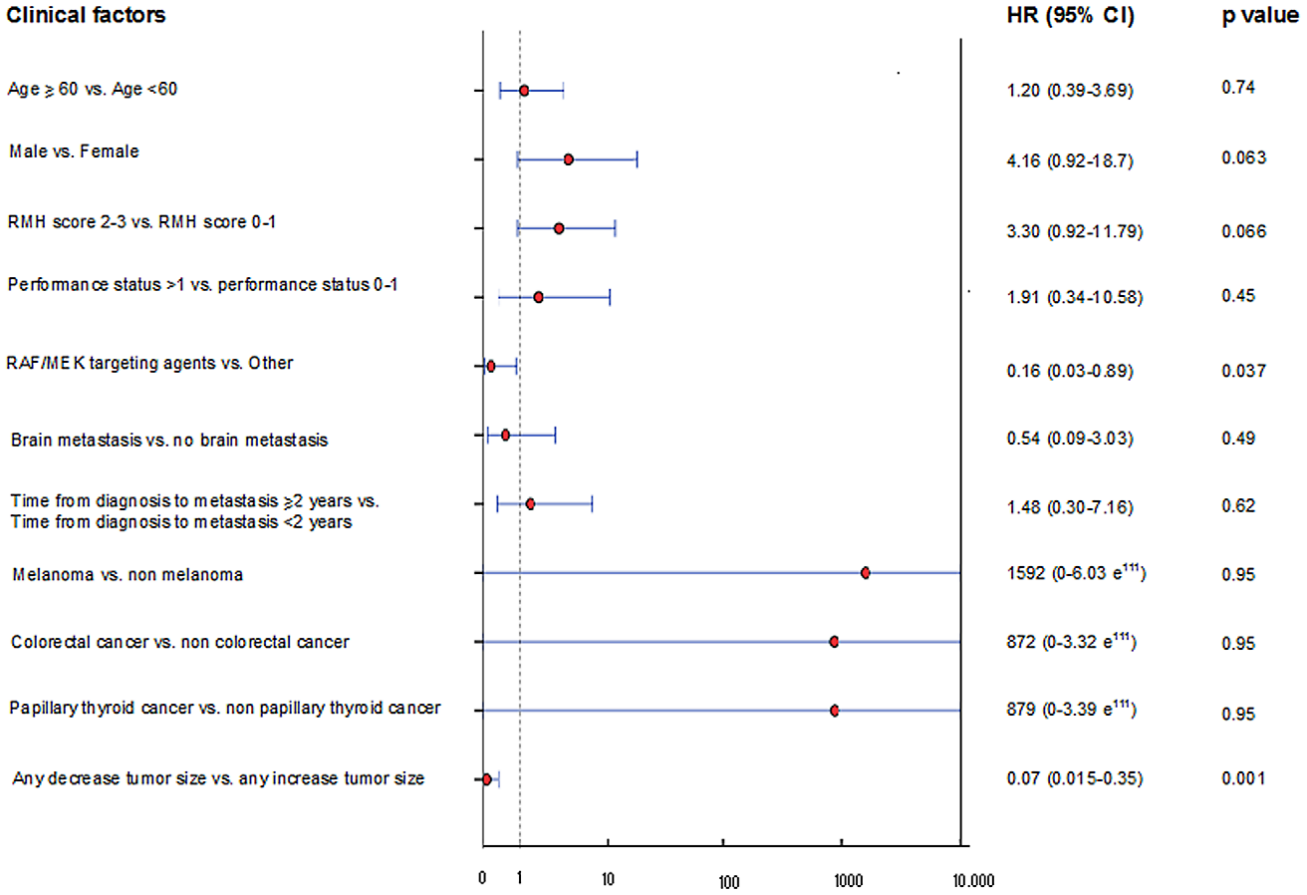
Forest plot summarizing the clinical factors affecting overall survival after referral and displaying their hazard ratio and 95% Confidence interval calculated by Cox proportional hazards regression model in patients with *mutBRAF* advanced cancer.

In multivariate analysis, the only two factors that predicted a superior OS after referral to the Phase I clinic in the *mutBRAF* group were treatment with any RAF/MEK targeting agents (HR 0.16, 95% CI, 0.03–0.89, p = 0.037) and any decrease in tumor size (RECIST measurement) on any phase I trial (HR 0.07, 95% CI, 0.015–0.35, p = 0.001) (Figure 4). Of note, the HR values of the following predictive factors “melanoma vs. non melanoma”, “colorectal cancer vs. non colorectal cancer” and “papillary thyroid cancer vs. non papillary thyroid cancer” are extremely high, compared to their HR calculated by univariate analysis (figure 4). This discrepancy could be explained by the difference in methodology used. Despite their high absolute values, this should be interpreted cautiously provided they don't have any statistical significance as demonstrated by a p value close to 1 and a 95% confidence interval that contains zero. Furthermore, their extremely wide 95CI% is indicative of the poor estimate of their value.

#### Univariate analysis in *wtBRAF* patients

A similar analysis was conducted in the *wtBRAF* group for the 104 patients referred. Univariate analysis revealed superior OS from referral associated with the following: RMH score of 0–1 compared to RMH score of 2–3 (50 months, 95%CI 6.4–93.3 vs. 6 months, 95%CI 2–10.3; p<0.001 and HR 2.94, 95%CI 1.56–5.56; p = 0.001), treatment with RAF/MEK targeting agents vs. treatment with any other agents or no treatment (51 months vs. 10 months, 95%CI 7.1–12.9; p = 0.014 and HR 0.32, 95%CI 0.12–0.83; p = 0.019), no brain metastases vs. brain metastases detected (15 months, 95%CI 0–34.3 vs. 7 months, 95%CI 3–10.3; p = 0.004 and HR 2.47, 95%CI 1.31–4.65; p = 0.005), non melanoma vs. melanoma (50 months vs. 10 months, 95%CI 6–13.9; p = 0.006 and HR 2.57, 95%CI 1.27–5.18; p = 0.008) and any decrease in tumor size vs. no decrease (50 months vs. 10 months, 95%CI 6.2–13.8; p = 0.006 and HR 0.32, 95%CI 0.13–0.75; p = 0.009) (Table 5).

**Table 5:** Univariate analysis of survival predictors after referral to phase 1 clinic in patients with *wtBRAF* advanced cancer.

Predictor	Median OS (95% CI)	N^4^	P value	HR	95% CI	P value
**Age≥60**	13.5 (7–20)	46	0.84	0.93	0.49–1.77	0.84
**Age<60**	11.1 (4.6–17.5)	58				
**Male**	10.3 (6.8–13.7)	69	0.21	1.56	0.76–3.21	0.22
**Female**	Unreached	35				
**RMH score** 1 **2–3**	6.2 (2–10.3)	36	**<0.001**	2.94	1.56–5.56	**0.001**
**RMH score 0–1**	49.8 (6.4–93.3)	68				
**Performance status >1**	Unreached	31	0.20	0.62	0.29–1.30	0.21
**Performance status 0–1**	9.5 (3.2–15.7)	73				
**RAF/MEK targeting agents**	50.6	22	**0.014**	0.32	0.12–0.83	**0.019**
**Other** 2	10 (7.1–12.9)	82				
**Brain metastasis**	6.7 (3–10.3)	33	**0.004**	2.47	1.31–4.65	**0.005**
**No Brain metastasis**	15.3 (0–34.3)	71				
**Time from diagnosis to metastases ≥2 years**	15.3 (0–37)	43	0.19	0.65	0.34–1.24	0.19
**Time from diagnosis to metastases <2 years**	9.5 (5.5–3.4)	61				
**Melanoma**	10 (6–13.9)	67	**0.006**	2.57	1.27–5.18	**0.008**
**Non melanoma**	49.8	37				
**Colorectal cancer**	8.9 (6.5–1.3)	20	0.74	1.13	0.53–2.40	0.74
**Non Colorectal cancer**	13.7 (4.9–22.6)	84				
**Papillary thyroid cancer**	Unreached	11	**<0.001**	0.027	0.001–0.68	**0.029**
**Non papillary thyroid cancer**	Unreached	93				
**Any decrease tumor size** 3	49.8	36	**0.006**	0.32	0.13–0.75	**0.009**
**Any increase tumor size**	10 (6.2–13.8)	44				

1 Royal Marsden Hospital (RMH) ^13^ prognostic score is determined as follows: 0 points, normal lactate dehydrogenase (LDH), albumin ≥3.5 g/dL, a ≤2 metastatic sites; 1 point- LDH>upper limit of normal, albumin <3.5 g/dL, >2 metastatic sites. Patients with 0–1 points had a good RMH score, and patients with 2–3 points had a poor RMH score.

2 Among the 149 patients with *wtBRAF*, 22 patients were treated with RAF/MEK targeting agents, 64 patients were treated with non RAF/MEK targeting agents, 18 patients never been enrolled in phase 1 trial after referral and 45 patients from melanoma department who were not referred to the phase 1 department the time of the analysis.

3 Patients who never had a restaging at the last follow-up or who never started on a phase 1 trial were excluded in the univariate analysis (N = 24).

4 Only patients who were referred to the phase 1 clinic were considered in this analysis (Overall survival from time of referral to the phase 1 clinic).

By excluding patients who did not get enrolled into a phase 1 trial after referral (18 patients total), we found that *wtBRAF* patients treated with RAF/MEK targeting agents has a trend towards improved survival after referral compared to *wtBRAF* patients treated with any other agents (51 months vs. 10 months, 95%CI 4.7–15.9; p = 0.052 and HR 0.39, 95%CI 0.14–1.04; p = 0.06)

#### Multivariate analysis in *wtBRAF* patients

**Figure 5:**
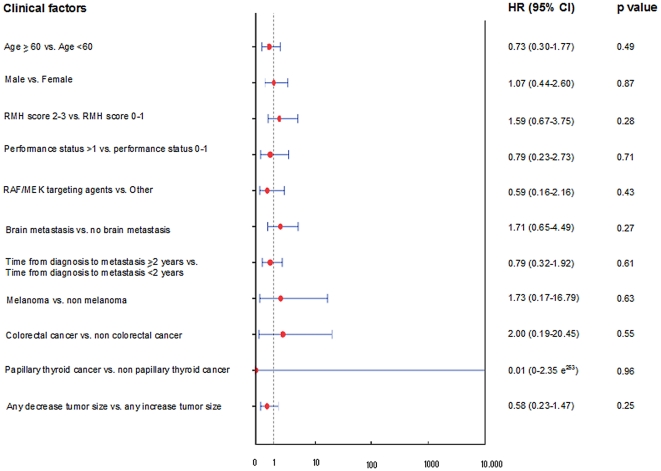
Forest plot summarizing the clinical factors affecting overall survival after referral and displaying their hazard ratio and 95% Confidence interval calculated by Cox proportional hazards regression model in patients with *wtBRAF* advanced cancer.

In the multivariate analysis, none of these factors was significantly associated with a better OS from referral (Figure 5).

### Characteristics of Melanoma Patients with *V600K BRAF* mutation

**Figure 6:**
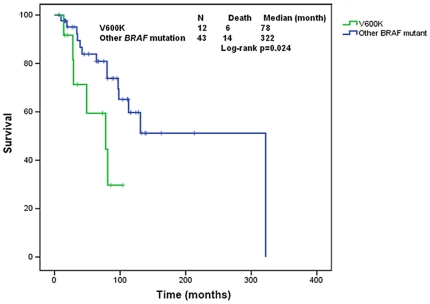
Kaplan Meier estimate of overall survival from time of diagnosis comparing patients with melanoma with *V600K BRAF* mutation vs. other *BRAF* mutations. Tic marks represent patients who were alive and censored at time of last follow up. (One patient for whom the time of diagnosis was unknown was excluded.)

We further investigated the behavior of *mutBRAF* melanoma with *V600K* substitution compared to other subtypes of *BRAF* mutation. (There were 13 patients with V600K mutations including 12 with melanoma and one with colorectal cancer). In the melanoma group, we compared patients with V600K *BRAF* mutations vs. non-V600K *BRAF* mutations (the vast majority being V600E). We found that V600K was associated with more brain (75% vs. 36.3%, p = 0.02) and lung metastases (91.6% vs. 47.7%, p = 0.007). (The single patient with colorectal cancer and V600K also had brain and lung metastases). V600K melanomas metastasized earlier (median time to metastasis = 19 months, 95%CI 0–49 vs. 53 months, 95%CI 33–72, p = 0.046), and were associated with a shorter OS from time of diagnosis (median 78 months, 95%CI 10–146 vs. 322 months, p = 0.024) (Figure 6).

We also compared the OS from diagnosis and from metastases between V600K melanoma vs. wtBRAF melanoma and it was not statistically different (P = 0.53 and 0.54, respectively).

Among the 13 patients with V600K *BRAF* mutation, eight received RAF/MEK targeting agents (of which one was colorectal cancer), three did not receive treatment (only best palliative care) and two received other targeting agents. There were two patients with stable disease of over four months, but no partial or complete remissions.

## Discussion

*BRAF* is one of the most frequently mutated protein kinase in cancer [14]. It has been reported in approximately 40 to 60% of melanoma, 40 to 70% of papillary thyroid carcinoma and 5 to 15% of colorectal cancer cases [6]. In this study we examined whether *mutBRAF* cancers exhibit any distinctive clinical features compared to *wtBRAF* cancer.

Overall, we found a higher frequency of women and younger patients with cancer harboring *BRAF* mutation compared to those without the mutation. These results are consistent with those in a smaller series (18 patients) with *mutBRAF* melanoma, in whom a higher frequency of patients younger than 60 and women was noted [15]. In our study, *mutBRAF* cancers were less likely to metastasize to the soft tissue, retroperitoneum and lungs and more likely to metastasize to the brain, suggesting that *mutBRAF* might affect the metastatic spread pattern of the disease. In the subset of patients with melanoma, the presence of *mutBRAF* is more likely associated with a time from diagnosis to distant metastasis beyond 2 years.

Kumar *et al* also reported a longer disease-free survival in *mutBRAF* melanoma compared to those without the mutation, although the difference was not statistically significant [16]. In a large Australian series of 207 patients with melanoma, *mutBRAF* was also associated with younger age; however, other clinical features, including time to metastases, response to chemotherapy and metastatic site were essentially indistinguishable [17]. In another report of 68 patients with melanoma, 30 of whom had mutant *BRAF*, an increase in the incidence of liver metastases was noticed in the *mutBRAF* group [18].

In *mutBRAF* colorectal cancer, Tran *et al* [19] observed a higher incidence of peritoneal disease and central nervous system involvement, but a lower incidence of lung metastases and a shorter OS from time of diagnosis. These data support our findings albeit without statistical significance, perhaps due to the small number of patients with colorectal cancer.

Some differences in the behavior of *mutBRAF* cancer were seen across histologies. Whereas *mutBRAF vs. wtBRAF* is associated with a trend towards longer OS from time of diagnosis in melanoma, OS from time of diagnosis tended to be shorter in colorectal cancer, albeit without reaching significance, perhaps due to the low number of patients. In multivariate analysis, *NRAS* and male gender were the only factors correlated with diminished OS from time of diagnosis in melanoma. Scoggins *et al* [20] found male gender associated with unfavorable survival in melanoma, however, gender difference did not appear to be a significant factor in a larger retrospective study [21]. Houben *et al* [22] showed a significantly decreased survival of *mutBRAF* metastatic melanoma, which is discordant with our data. In our population, the longer disease-free interval and possibly the introduction of new targeted therapy against *mutBRAF* melanoma [6] might explain, at least in part, the improvement in overall survival from time of diagnosis favoring the *mutBRAF* group.

*mutBRAF* was in general mutually exclusive with the presence of *mutRAS* (*KRAS*, *NRAS*). Interestingly, however, we observed one patient with a concomitant *NRAS* and *BRAF* mutation. This observation might be explained by different clones of cancer cells inside the tumor with distinct dual mutations. A similar finding was previously reported in familial melanoma, with *CDKN2A* as well as *BRAF* and *NRAS* mutations [23]. It is believed that *BRAF* and *NRAS* mutations can coexist within the same melanoma but not at the single-cell level [24].

We also examined the response to best standard systemic treatment. In melanoma, we noted that there was a trend towards a shorter PFS among patients with *mutBRAF* but this did not reach statistical significance. Findings in the published literature are conflicting. Joseph *et al* did not find an impact of *NRAS* or *BRAF* mutational status on response to high dose interleukin-2 in metastatic melanoma [25]. Similarly, Chang *et al* [18] reported no difference in response rate to systemic treatment between *mutBRAF* and *wtBRAF* melanoma. In another series by Kumar and colleagues, patients with *mutBRAF* melanoma had a diminished response to therapy [16]. In colorectal cancer, we showed that *mutBRAF* was independently associated with a shorter PFS on best standard systemic therapy. Our observations are consistent with those of others which have demonstrated that *mutBRAF* is an adverse predictor in colorectal cancer [26]–[29]. Further, it has been recently suggested that *mutBRAF* may also predict resistance to cetuximab-based regimens, though it is still unclear whether the mutation is indeed a predictor of resistance or a prognostic marker for a subgroup that simply does worse [30]–[32]. *mutBRAF* has also been linked to a shorter PFS on standard chemotherapy in few studies [33], although these findings have been disputed by others [28]. In regard to thyroid cancer, many series have demonstrated high-risk features associated with *mutBRAF* in papillary thyroid carcinoma. Xing *et al* [34] also reported an association between *mutBRAF* and the rate of tumor recurrence, though these results have not been confirmed by other studies [35]. The number of patients with papillary thyroid cancer in our study precluded making conclusions on this issue.

Our study demonstrated that treatment with RAF/MEK targeting agents and initial tumor shrinkage are independent factors associated with improved survival in patient with mutant *BRAF*. These findings support data from a series of published individual studies with molecules including but not limited *to* PLX4032, GSK2118436 (BRAF inhibitor), and GSK 1120212 (MEK1/2 inhibitor) in *mutBRAF* cancer [3]–[6]. Multivariate analysis conducted on patients with *wtBRAF* found no association between treatment with RAF/MEK targeting agents and survival.

Interestingly, we identified *V600K BRAF* mutation as a prognostic factor associated with more aggressive behavior in metastatic melanoma. Indeed, *V600K* associated with more brain metastases, shorter time for both disease-free survival and OS from diagnosis, and a trend towards a shorter OS following metastases in comparison to melanoma with other types of *mutBRAF* (Figure 6). Because of the small number of patients, it is unclear as to how this would impact *BRAF*- targeted therapies, other than the fact that treatment that penetrates the brain might be needed.

Our analysis has limitations: (i) the small number of patients in each histologic group; (ii) the absence of randomization in regard to the PFS and overall survival data; (iii)the possibility of selection bias based on treatment choice; (iv) selection bias because we only analyzed patients with metastatic disease and cannot therefore ascertain the behavior of patients whose disease never metastasized; (v) the retrospective nature of the study; and (vi) the fact that multiple tests were analyzed for significance. Taken together, this study must therefore be considered exploratory. Even so, several observations that merit further investigations emerge. First, some clinical features appear to differ between histologies despite the presence of *BRAF* mutation. For instance, patients with colorectal cancer and *BRAF* mutation showed a trend towards poor overall survival from diagnosis while, in patients with melanoma, the presence of a *BRAF* mutation was associated with a trend towards better survival. Other factors, including a higher frequency of women and younger patients with cancer harboring *BRAF* mutation compared to those without the mutation, as well as a lower likelihood to metastasize to the soft tissue, retroperitoneum and lungs was seen across histologic groups, albeit not always in a statistically significant manner. Overall, the only independent factors predicting survival in *BRAF*- mutant patients in our clinic was treatment with any RAF/MEK axis targeting agent and any initial tumor regression. Of interest, our preliminary data also suggest that the site of mutation may be important, since the subgroup with *V600K BRAF* mutation (as opposed to *V600E*) was associated with more brain metastases, and shorter time for both disease-free and overall survival from diagnosis in melanoma. These data support a role for *BRAF* as a driver mutation that influences phenotype and that provides a druggable target for patients with cancer.
